# The quail anatomy portal

**DOI:** 10.1093/database/bau028

**Published:** 2014-04-07

**Authors:** Avnika A. Ruparelia, Johanna E. Simkin, David Salgado, Donald F. Newgreen, Gabriel G. Martins, Robert J. Bryson-Richardson

**Affiliations:** ^1^School of Biological Sciences, Monash University, Melbourne, Victoria 3800, Australia, ^2^The Murdoch Childrens Research Institute, The Royal Children's Hospital, Flemington Road, Parkville, Melbourne, Victoria 3052, Australia, ^3^Australian Regenerative Medicine Institute, Monash University, Clayton, Victoria 3800, Australia, ^4^Aix Marseille Université, Inserm, GMGF UMR_S 910, 13385 Marseille, France, ^5^Instituto Gulbenkian de Ciencia, Rua da Avenida Grande 6, 2780-156 Oeiras, Portugal and ^6^Centre for Environmental Biology, Faculdade de Ciencias da Universidade de Lisboa, Campo Grande, 1749-016 Lisbon, Portugal

## Abstract

The Japanese quail is a widely used model organism for the study of embryonic development; however, anatomical resources are lacking. The Quail Anatomy Portal (QAP) provides 22 detailed three-dimensional (3D) models of quail embryos during development from embryonic day (E)1 to E15 generated using optical projection tomography. The 3D models provided can be virtually sectioned to investigate anatomy. Furthermore, using the 3D nature of the models, we have generated a tool to assist in the staging of quail samples. Volume renderings of each stage are provided and can be rotated to allow visualization from multiple angles allowing easy comparison of features both between stages in the database and between images or samples in the laboratory. The use of JavaScript, PHP and HTML ensure the database is accessible to users across different operating systems, including mobile devices, facilitating its use in the laboratory.The QAP provides a unique resource for researchers using the quail model. The ability to virtually section anatomical models throughout development provides the opportunity for researchers to virtually dissect the quail and also provides a valuable tool for the education of students and researchers new to the field.

**Database URL**: http://quail.anatomyportal.org

(For review username: demo, password: quail123)

## Introduction

The Japanese quail (*Coturnix coturnix japonica*) has been extensively used in quail–chick chimera studies to investigate the development of a diverse range of cell types (1). Recently it has become increasingly used as a model system in its own right (2, 3) because of its relatively small size, high egg productivity, low cost and significantly shorter time to maturity compared with chicken (4). The recent availability of transgenic quail (4), facilitating *in *vivo imaging and greatly extending the usefulness of this model (5), and the release of a draft genome sequence (6) are likely to result in further use of this model system in the future.

Although anatomical resources exist for the chicken model, developmental differences mean these cannot be applied unreservedly to the quail. A quail staging guide based on external features, comparable with the chicken staging table of Hamburger and Hamilton (7), has been released (8). For detailed anatomical models, previous efforts have used magnetic resonance imaging to generate three-dimensional (3D) images of quails between embryonic day 5 and 10 for download (9). To expand the limited resources available for this emerging model, we have developed the Quail Anatomy Portal (QAP; http://quail.anatomyportal.org). This presents a complete 3D staging generated series using high-resolution 3D Optical Projection Tomography (OPT). The staging series contains 22 virtual embryos ranging from embryonic day 1 (E1) to E15 that can be viewed and rotated to assist in staging of samples. Furthermore, the 3D data sets can be virtually sectioned to visualize the internal structures for 20 stages from E1 to E11 and are available for download.

## Construction and content

### Three-dimensional models

Quail embryos staged from E1 to E15 (1.5 days before hatching) were incubated at 38°C, collected and fixed in 4% parafomaldehyde overnight. All embryos were dehydrated into 100% methanol, and embryos older than 4 days were bleached by immersion in bleaching solution (Methanol: DMSO: 30% H_2_O_2_ in a 4:1:1 ratio) to remove pigmentation. The embryos were then prepared and mounted for OPT as previously described (10, 11). OPT for samples from E1 to E10 was carried out using the Bioptonics 3001 OPT scanner using sample autofluorescence (emission filters: 475/40 nm or 610/75 nm), under exposure to fluorescent light (excitation filters: 420 nm longpass or 545/30 nm). For the E9 and E10 stages, two scans of each sample were acquired, covering the top and bottom half of the sample, respectively, and the two 3D models joined using the ‘Pairwise stitching’ plug-in offered by Fiji (12). Late-stage embryo OPT data sets were acquired on a custom-built OPT setup (13) using a large cuvette, and images of fluorescence (510 nm LP filter, excitation illumination with a 470 nm LED source; embryos E10.5–E13), or in transmitted light mode with diffused white light (E15). For each sample, 1600 images were acquired over a 360° rotation. The image analysis software Fiji (14) was used to correct for ‘hot’ pixels and photo-bleaching of the sample through the use of a rank filter to remove bright pixel outliers and multiplying pixel intensity values by a correction factor determined by fitting an exponential curve to the decreasing mean fluorescence intensity over the image series. Skyscan's NRecon software was used to carry out volumetric reconstruction. The reconstructed data sets were rotated in 3D to ensure consistency in orientation and virtually sliced in each of the three standard planes (sagittal, coronal and transverse) using Fiji. Note that to allow for easier identification of internal structures, where necessary, extra-embryonic tissue was virtually trimmed out from each of the data sets. For each model, standard deviation projections for the sagittal and coronal sections and false coloured 3D volume renderings were generated, using Fiji and the volume rendering software Drishti (15), respectively. Limited internal detail was resolved in the E13 and E15 samples because of the absorption of light by these larger specimens, and as a result, these are only presented in the staging tool.

### Database and user interface

The system was based on the previously described Zebrafish Anatomy Portal (16). The user interface and database have both been designed to display and store 3D data sets and anatomical information at multiple stages of development. Furthermore, the database has been designed to allow the collection of multiple different organisms. We have designed the QAP with a three-tier architecture (user browser, webserver and database server). The front end of the Web site is developed with web scripting languages such as PHP, HTML and JavaScript for data access, manipulation and interactivity. The user interface was generated using PHP and JavaScript including the OpenLayers (http://openlayers.org) library to display and manipulate the images. The PHP and JavaScript-based interface ensure compatibility across different operating systems and browsers and does not require any additional software or plug-in to be installed. The staging tool was created using the jQuery Reel plug-in (http://jquery.vostrel.cz) to allow interactive visualization of the Drishti volume renderings. The data are gathered within a PostgreSQL database (version 9.08) and queried via PHP scripts on the webserver. Section images are stored within the core database as PostgreSQL binary large objects. The relational schema has been designed to store individual sections from the three orthogonal planes independently for each developmental stage. We have also integrated the feature to allow multiple samples at the same developmental stage. This feature would allow future expansion to include and compare samples for male, female, different genetics strains or even experimental conditions at the same stage.

To facilitate the growth and management of the database, we developed a series of PHP scripts. Image data are supplied as a series of section images for each of the three views. To ensure consistency the scripts change the file names, without affecting the plane order, check the integrity of the data (no missing or duplicated planes) and check whether the dimensions of all the images match and would therefore form the same 3D object. Once the data have been verified, another script uploads the image data and associated organism and staging data.

## Using the QAP

**Figure 1.**
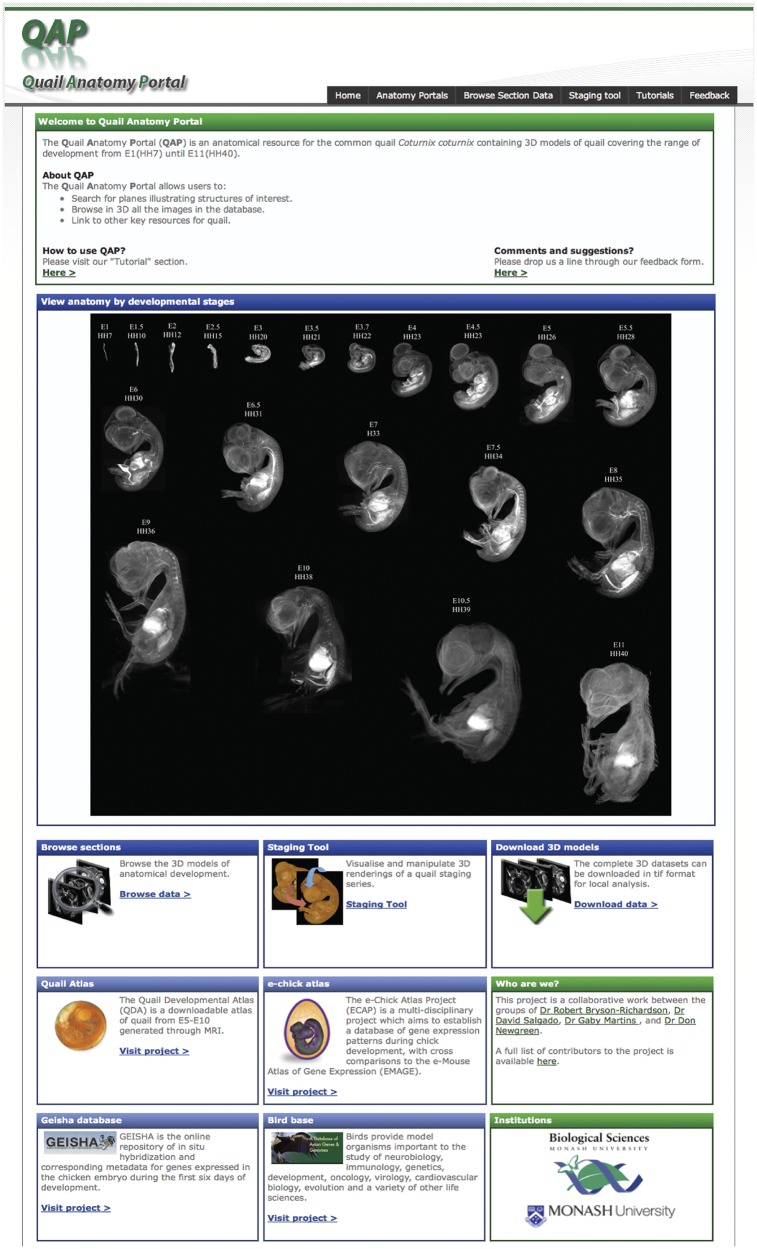
The QAP home page. The home page facilitates rapid identification of the required information by the user. The section data for each stage can be browsed by following the link in the toolbar or in the browse section box. However, the desired stage may also be accessed directly by clicking on the 3D rendered images in the staging series image. The home page also provides access to the staging tool and download area. In addition to the resources available in the QAP the home page also provides links to other related resources.

The homepage (Figure 1) provides access to a section browser, staging tool and the 3D models for download. The toolbar, present on all pages of the site, provides rapid access to the tools available and the tutorial and feedback areas. The section browser can be accessed via the tool bar or the feature box on the main page. A staging series consisting of 3D rendered images of each model in the data set is presented. To facilitate rapid access to a specific stage in the section browser, clicking on any of the quail images will open the section browser at the appropriate stage.

**Figure 2.**
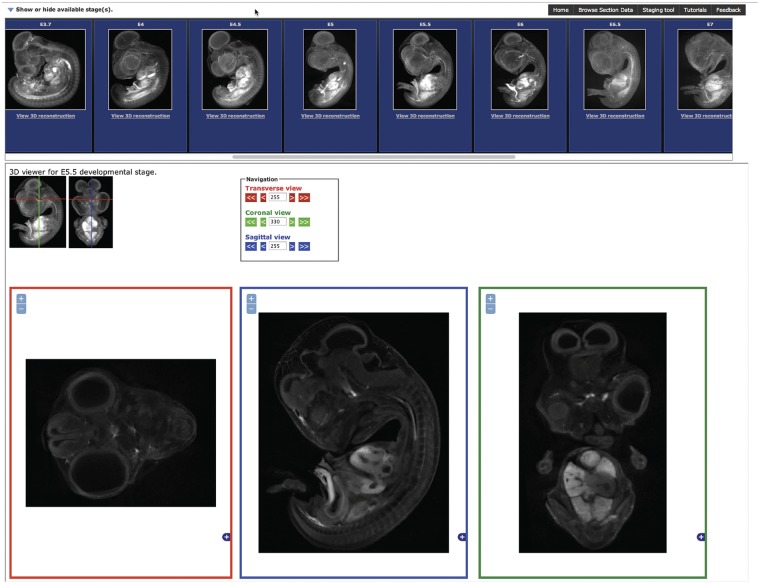
The Section Browsing Tool. The stage of interest can be selected by clocking on the rendered images in the top panel. Once a stage has been selected, the three section views, transverse, sagittal and coronal will be displayed. The displayed section can be updated by clicking on the 3D rendered images and the image size adjusted using the plus and minus buttons in each window.

The section browser interface (Figure 2) allows the user to select the stage required by clicking on the standard deviation projection images presented at the top of the page. Once a stage is selected, three section views are displayed corresponding to transverse, sagittal and coronal sections of the embryo. The boxes for section display will resize to fit the size of the window available. Once a stage has been selected, the stage selection panel can be hidden by clicking on the arrow in the top left to allow the complete window area to be used for section display. The section displayed can be updated by clicking on the 3D representation or on the arrow buttons in the navigation area providing an intuitive user interface to browse the section data.

**Figure 3.**
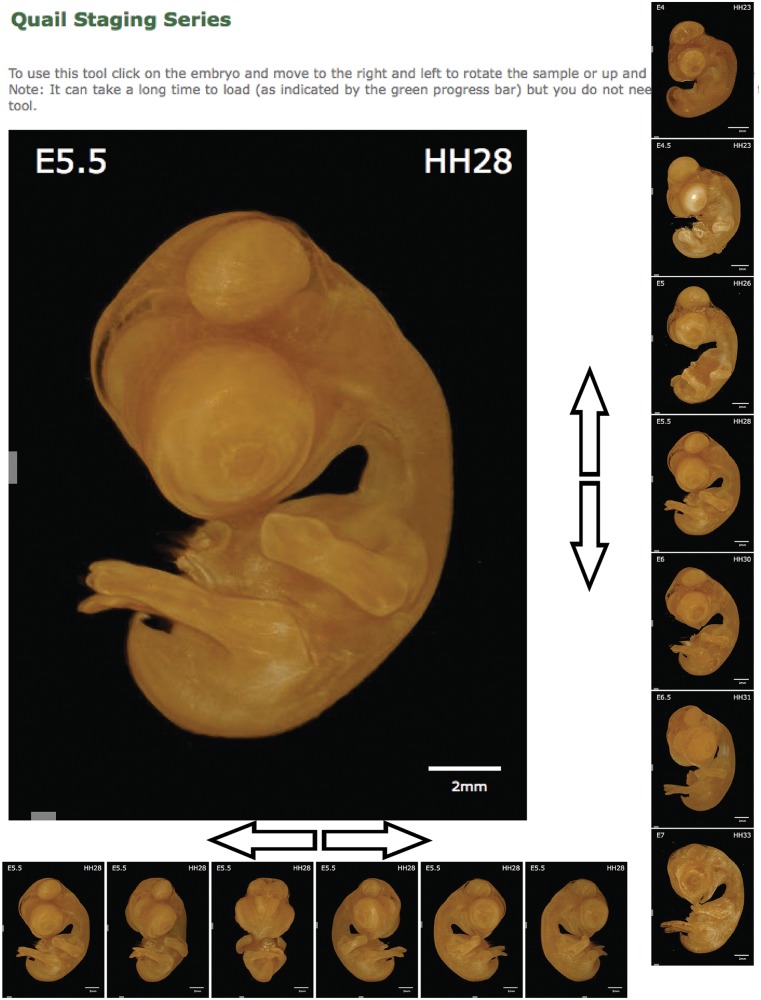
Quail Staging Tool. The staging tool displays renderings of every stage in the database viewed from multiple angles. The sample displayed can be rotated to allow observation of the desired feature by clicking on the image and moving the cursor left or right. The stage displayed can be similarly altered by clicking on the image and moving the cursor up or down. The stage of the sample is presented as both embryonic day (E) and Hamburger Hamilton stage (HH).

The staging series image presented on the home page provides both a method to select the stage required and a guide to staging quail embryos. However, to make full use of the 3D data sets generated in the staging of quail samples, we generated a staging tool. The staging tool (Figure 3) is designed to facilitate accurate staging of quail embryos allowing direct comparison with an image or sample under the microscope. A 3D volume rendering of an embryo is displayed on screen together with a scale bar and the stage of the sample both by embryonic day (E) and Hamburger Hamilton stage (HH); by clicking on the image and moving left or right, the angle of the embryos is rotated, allowing its position to be changed to match the position of the embryo you wish to stage. By clicking and moving the mouse up or down, the stage of the embryo is altered, allowing the changes in features between stages to be analysed. By allowing easy comparison of stages and manipulation of the viewing angle to match the users sample, the staging tool provides a significant advantage over 2D staging series. On mobile devices or computers with restricted bandwidth, the staging tool may not be fully downloaded immediately, and therefore, the progress is indicated by a green bar at the bottom of the tool. The tool does not need to be completely downloaded before use, but the number of angles and stages available will increase incrementally until the complete data set is downloaded. To further aid in the use of the tools available, an annotated tutorial video is available demonstrating the use of both the staging tool and the section browser.

The use of PHP, HTML and JavaScript to develop the section browser and staging tool not only ensures support across many browsers and platforms but also allows their use on mobile devices such as phones and tablets, conveniently allowing their use in the laboratory while examining samples. The creation of the QAP and the development of improved data visualization tools significantly extends the utility and capability of the previous anatomy portal we created, which was limited to zebrafish data (14). Importantly, the developments required for the creation of the QAP now provide the ability to continue to extend the anatomy portal resource (http://anatomyportal.org) to include data from additional species. It is our intention to continue to add additional species to the database, providing not only a resource for researchers using different organisms but ultimately, as more species are added, a resource for comparative anatomy and evolutionary studies.

## Conclusions

The QAP provides a unique anatomical resource and staging tool. The database provides section data and a staging tool, covering embryonic development from E1 to E15. The significant improvement in image resolution and the range of stages covered over existing resources makes this the most complete quail anatomical atlas available to date, providing an important tool for quail researchers and others interested in quail development. The expansion of the anatomy portal to include multiple species also establishes a platform that will facilitate development of similar resources for other species of interest and ultimately a resource for comparative anatomy.
